# Efficacy of e-cigarettes for smoking cessation in populations with psychiatric and/or substance use problems: A secondary analysis of a randomized controlled trial

**DOI:** 10.18332/tpc/199473

**Published:** 2025-02-03

**Authors:** Stéphanie Baggio, Philip Bruggmann, Anna Schoeni, Nazanin Abolhassani, Kali Tal, Susanne Pohle, Anja Frei, Jean-Paul Humair, Isabelle Jacot-Sadowski, Janine Vetsch, Luca Lehner, Anna Rihs, Laurent Gétaz, Aurélie Berthet, Moa Haller, Mirah Stuber, Julian Jakob, Reto Auer

**Affiliations:** 1 Institute of Primary Health Care (BIHAM), University of Bern, Switzerland; 2 Laboratory of Population Health (#PopHealthLab), University of Fribourg, Switzerland; 3 Arud Centre for Addiction Medicine, Zurich, Switzerland; 4 Institute of Primary Care, University Hospital of Zurich, Zurich, Switzerland; 5 Lung Center, Kantonsspital St. Gallen, St. Gallen, Switzerland; 6 Epidemiology, Biostatistics and Prevention Institute, University of Zurich, Zurich, Switzerland; 7 Department of Primary Care Medicine, University Hospitals of Geneva, Geneva, Switzerland; 8 Centre for Primary Care and Public Health, University of Lausanne, Lausanne, Switzerland; 9 Department of General Internal Medicine, Inselspital, Bern University Hospital, University of Bern, Bern, Switzerland; 10 Division of Prison Health, Geneva University Hospitals, Geneva, Switzerland; 11 Department of Paediatrics, Inselspital, Bern University Hospital, University of Bern, Bern, Switzerland

**Keywords:** smoking cessation, disparities, mental disorders, randomized controlled trial

## Abstract

**INTRODUCTION:**

People with psychiatric and substance use disorders are more likely to smoke and less likely to quit than smokers in the general population. We evaluated the efficacy of e-cigarettes for abstinence from tobacco smoking in people with psychiatric and substance use problems.

**METHODS:**

We analyzed data collected in the larger ‘Efficacy, Safety, and Toxicology of ENDS as an Aid for Smoking Cessation’ (ESTxENDS) trial (n=1246): the intervention group received e-cigarettes and e-liquids, plus standard-of-care smoking cessation counseling (SOC) for 6 months; the control group received SOC and a voucher. The primary outcome was biochemically validated continuous self-reported abstinence at 6 months; secondary outcomes included 6-month and 7-day self-reported abstinence. We calculated adjusted relative risks (ARR) for two subsamples meeting these conditions at the baseline visit: 1) psychotropic medication use; and 2) problematic substance or polysubstance use.

**RESULTS:**

Among the participants using psychotropic medications (n=239), the ARR for validated abstinence was 2.62 (95% CI: 1.40–4.90) in the intervention group versus the control group, 2.95 (95% CI: 1.72–5.07) for 6-month and 2.96 (95% CI: 1.92–4.55) for 7-day self-reported abstinence, while among participants with problematic substance or polysubstance use (n=818), the ARR was 1.57 (95% CI: 1.20–2.04), 1.42 (95% CI: 1.15–1.74), and 1.53 (95% CI: 1.31–1.79), respectively.

**CONCLUSIONS:**

Adding e-cigarettes to standard-of-care counseling increased the likelihood that participants with psychiatric and substance use problems would abstain from smoking, but larger studies should test the efficacy and safety of smoking cessation interventions in this often-marginalized population.

## INTRODUCTION

Smoking contributes to health inequalities for people with psychiatric and substance use problems^1^. Though smoking rates have declined among the general population in Western countries, the rate of decline is far less among those with psychiatric and substance use problems^2,3^. The slower rate of decline is a concern because people with these problems are consistently more likely to smoke, less likely to quit smoking, and more likely to have tobacco-related health problems than the general population^2,4-6^. They may also be more likely to believe that smoking relieves their symptoms or helps with social interactions^4,7^, to rank smoking cessation well below their other health-related issues^7^ and to have negative attitudes towards the healthcare professionals who offer smoking cessation counseling or treatment^8^.

Despite research that demonstrates smoking is a modifiable risk factor for excess mortality in people with psychiatric disorders^3,5,9^, few smoking cessation efforts have focused on meeting their needs^2^. Given the paucity of evidence, we need to know if e-cigarettes, an increasingly popular method of smoking cessation that research suggests is effective and safe for smoking cessation in the general population^10,11^, are also safe and effective for those with psychiatric and substance use problems^4,12^.

We thus evaluated the efficacy of adding e-cigarettes to standard care smoking cessation counseling versus standard care alone, by measuring abstinence from tobacco smoking at 6 months in people with psychiatric and substance use problems. To estimate the benefits of e-cigarettes for smoking cessation in this population, we used data from a large randomized controlled trial (RCT) with minimal exclusion criteria.

## METHODS

### Design and setting

This study was a secondary analysis of the Efficacy, Safety, and Toxicology of Electronic Nicotine Delivery Systems as an aid for smoking cessation (ESTxENDS) trial, an open-label, multicentric RCT (ID: NCT03589989) conducted in five study sites in Switzerland^10^. ESTxENDS was designed to compare, at 6 months after the quit date, the efficacy and safety of e-cigarettes as a smoking cessation aid when added to standard care. Participants were recruited between July 2018 and June 2021 via advertisements in the lay press, social media, public transport, and healthcare facilities. Local ethics committees for each study site approved the trial. The main results are published elsewhere^10^.

### Participants

Participants were eligible if they met the following inclusion criteria: 1) aged ≥18 years; 2) smoked ≥5 cigarettes/day in the last 12 months; and 3) willing to quit smoking within 3 months. Exclusion criteria included: 1) pregnancy or breastfeeding; 2) use of nicotine replacement therapy or smoking cessation drugs within the last 3 months; 3) regular use of e-cigarettes or tobacco heating systems in the last 3 months; 4) inability to understand study processes; and 5) could not attend the follow-up at 6 months. The study did not exclude participants with somatic or psychiatric diseases.

### Intervention

Participants were randomized at a 1:1 ratio into two study groups. The control group received standard smoking cessation counseling based on cognitive behavioral therapy, motivational interviews, and shared decision-making^10^. Participants were counseled and encouraged to use nicotine replacement therapy and smoking cessation. They received a voucher of CHF 50 (10 Swiss Francs about US$10.9) that they could spend on anything they wanted, including nicotine replacement therapy. The intervention group received the same standard smoking cessation counseling as the control and received two e-cigarette starter kits and free, ad libitum e-liquids for 6 months.

### Outcomes

*Primary outcome*

This was continuous biochemically validated abstinence from tobacco smoking at 6 months based on self-report, biochemically validated by anabasine level (<3 ng/mL) in urine^13-15^. If anabasine level was unavailable, abstinence was biochemically validated with exhaled carbon monoxide (≤9 ppm)^15^.

*Secondary outcomes*

These were: 1) Self-reported continuous abstinence from tobacco smoking at 6 months without validation; and 2) Self-reported abstinence in the 7 days preceding their visit, with and without validation.

### Groups

We defined two subsamples of participants according to baseline characteristics.

*Subsample of participants with psychiatric problems*

Psychiatric conditions were proxied by the use of psychotropic medications^16^. Participants were considered to have psychiatric problems if they reported using any of the following medications at baseline: benzodiazepine, antidepressant, hypnotic, sedative, or antipsychotic.

*Subsample of participants with substance use problems*

We included: 1) participants who reported problematic alcohol use at baseline, defined as scores of ≥3 points for women and ≥ 4 points for men on the Alcohol Use Disorders Identification Test-Concise (AUDIT-C)^17^; 2) participants who reported problematic cannabis use during the 6 months before baseline, defined as a score ≥8 on the Cannabis Use Disorder Identification Test-Revised (CUDIT-R)^18^; and 3) polysubstance use at baseline, defined as the use of at least two illicit substances during the 6 months before baseline. Participants could be included in both groups if they had psychiatric problems and substance use problems.

### Other measures

*Sociodemographics*

We assessed age, sex, marital status (single, divorced or widowed vs married or registered partnership), education level (mandatory school, secondary education, tertiary education), and working situation (employed/in training vs unemployed).

*Smoking characteristics*

We assessed cigarettes/day, whether participants have stopped cigarette smoking for at least 24 hours, and the Fagerström test for nicotine dependence (score range: 1–10; higher score indicates greater dependence)^19^. At 6 months, we also collected information on exposure to e-cigarettes, tobacco, and nicotine replacement therapy.

### Statistical analysis

The sample size calculation for the ESTxENDS trial was based on the primary outcome^10^. We did not perform a formal sample size calculation for this secondary analysis. We first used inverse probability of treatment weighting (IPTW) and stabilized weights to account for the potential lack of randomization balance in each subsample. The main advantage of IPTW is that it allows adjustment for covariates between groups by creating a pseudo-population in which exposure is independent of the measured confounders. Unlike regression, which adjusts for confounding through model coefficients, IPTW directly addresses imbalances in the distribution of confounders between groups. Unlike propensity score matching, it uses the entire sample and thus retains statistical power. This method is also very flexible, allowing IPTW to be combined with other types of weights, such as attrition weights (see below). We fitted a logistic regression with the exposure (presence/absence of psychiatric or substance use problems) as the outcome and covariates as factors. In both subsamples, covariates included age, gender, marital status, education level, work status, age first used cigarettes, cigarettes/day, history of quit attempts, and Fagerström test for nicotine dependence score. In the subsample with psychiatric problems, we included at-risk alcohol use, problematic cannabis use, and polysubstance use; in the subsample with substance use problems, we included the use of psychotropic medications. Covariates were selected using a directed acyclic graph, which helped to visualize potential confounders based on previous literature on factors confounding smoking cessation^20,21^. The fitted values from this model constituted the denominator of the IPTW.

We then fitted a second saturated logistic regression model that included only exposure as the outcome. Fitted values constituted the numerator of the IPTW, which we calculated separately for the subsamples with psychiatric problems and substance use problems. These weights created a pseudo-population in which exposure was not associated with covariates. To account for the potential selection bias due to attrition, we used inverse probability of censoring weights (IPCW)^22,23^. The principle was the same as IPTW, except for censoring or not censoring the outcome variable of the logistic regression models based on the presence or absence of attrition. We used stabilized weights because they preserve the original sample size and provide more appropriate variance estimates while reducing the impact of extreme weights. We then multiplied stabilized IPCW and IPTW to calculate the final weights. We truncated weights at the 99th percentile^24^.

Second, we used descriptive statistics to summarize baseline characteristics, including means and standard deviations, or percentages and frequencies. There were 98 missing values at baseline (at-risk alcohol: 7; CUDIT-R: 12; and polysubstance use: 98 missing values), which we imputed with simple imputation. Simple imputation was chosen over multiple imputations because multiple imputations created problems with covariate balance when performing balance diagnostics for the IPTW across multiple imputed datasets. We present descriptive statistics with IPTW to determine imbalances between the groups, and we report weighted standardized mean differences (SMDs) between intervention and control groups for variables accounted for in the IPTW. We also calculated unweighted descriptive statistics for nicotine exposure at 6 months.

Third, we analyzed separately the subsamples of participants with psychiatric problems and with substance use problems. We did not present stratified analyses for groups without psychiatric problems because our inability to reliably assess the absence of psychiatric problems could have caused information bias and because our study was not powered to make such comparisons. For both the primary and secondary outcomes, we used log-binomial regression models with IPTW and IPCW to compute the relative risks of smoking status at 6 months. We report 95% confidence intervals (CI). We present crude analyses and analyses adjusted for baseline covariates to improve precision and power^25^. We added the covariates of at-risk alcohol use, problematic cannabis use, and polysubstance use in the subsample with psychiatric problems and added the use of psychotropic medications in the subsample with substance use problems. Additionally, we examined sex-specific effects by adding an interaction term between the treatment group and participant sex for the primary outcome. For our sensitivity analysis, we considered participants without validation and drop-outs to be non-abstainers. We also conducted subgroup analyses among participants using different types of psychotropic medication to explore potential heterogeneity within this subsample. We conducted intention-to-treat analyses in Stata 18 with a two-tailed 0.05 significance level.

## RESULTS

Of smokers screened, we randomized 1246/2027^10^. Of the 2027, 13 could not understand instructions and were thus ineligible since they were likely to have severe mental health problems or impaired decision-making capacity. Of eligible participants, we excluded two because they had no data on relevant variables for this secondary analysis (either screening failure in the intervention group or withdrawal in the control group). We derived IP weights from the final sample, which included 1244 participants. The subsample with psychiatric problems (identified by psychotropic medication use) included 239 participants (n=117 in the intervention group, and n=122 in the control). The subsample of participants with substance use problems included 818 participants (n=399 in the intervention group, and n=419 in the control). In Table 1, we report weighted descriptive statistics using IPTW. Groups were balanced on most variables; the least balanced variables were marital status (SMD=0.29) and at-risk alcohol use (SMD=0.21) in the subsample with psychiatric problems. In the subsample of participants with psychiatric problems, 61.5% also had substance use problems. In the subsample of participants with substance use problems, 18.0% also had psychiatric problems. Descriptive statistics of the types of psychotropic medications used are reported in Table 2.

**Table 1:** Weighted descriptive statistics for the two subsamples of the randomized controlled trial ESTxENDS at baseline, Switzerland, 2019–2021

*Characteristics*	*Subsample with psychiatric problems* *(N=239)*			*Subsample with substance use problems* *(N=818)*		
	*Intervention* *% (n)*	*Control* *% (n)*	*SMD*	*Intervention* *% (n)*	*Control* *% (n)*	*SMD*
**Total**	117	122		399	419	
**Site**						
Bern	30.4 (35)	36.2 (44)		35.9 (143)	36.1 (151)	
Geneva	18.5 (22)	22.6 (28)		24.0 (96)	25.2 (106)	
Lausanne	11.0 (13)	19.3 (24)		12.6 (50)	12.7 (53)	
St. Gallen	16.7 (20)	7.7 (9)		14.7 (59)	12.9 (54)	
Zurich	23.3 (27)	14.2 (17)		12.8 (51)	13.0 (55)	
**Gender**						
Women	48.4 (57)	47.2 (58)		47.3 (189)	47.0 (197)	
Men	51.6 (60)	52.8 (64)	0.024	52.7 (210)	53.0 (222)	0.006
**Age** (years), mean (SD)	40.4 (13.5)	42.5 (12.5)	0.164	39.6 (13.6)	41.6 (13.9)	0.151
**Marital status**						
Single/divorced/widowed	67.9 (79)	80.0 (98)		72.7 (305)	73.4 (293)	
Married/registered partnership	32.1 (38)	20.0 (24)	**0.285**	27.3 (114)	26.6 (106)	0.017
**Education level**						
Primary	7.2 (8)	7.0 (9)		7.3 (29)	8.0 (33)	
Secondary	46.7 (55)	41.4 (50)	0.025	48.3 (193)	42.7 (179)	0.079
Tertiary	46.1 (54)	51.7 (63)	0.117	44.4 (177)	49.3 (207)	0.114
**Work status**						
Employed/training	74.6 (87)	76.6 (93)		71.2 (284)	73.4 (307)	
Unemployed	25.4 (30)	23.4 (29)	0.040	28.8 (115)	26.6 (112)	0.047
**Smoking status**						
Age first used cigarettes, mean (SD)	17.5 (3.8)	17.5 (4.5)	0.006	17.3 (3.9)	17.4 (3.5)	0.013
Cigarettes/day, mean (SD)	16.4 (8.5)	16.2 (7.5)	0.023	16.8 (8.1)	17.0 (7.9)	0.023
Fagerström score, mean (SD)	4.4 (2.3)	4.0 (2.5)	0.182	4.4 (2.3)	4.3 (2.3)	0.033
**Tried to quit smoking**						
No	12.7 (15)	16.7 (20)		13.8 (55)	15.2 (64)	
Yes	87.3 (102)	83.3 (102)	0.113	86.2 (344)	84.8 (355)	0.041
**At-risk alcohol use**						
No	47.1 (55)	36.6 (45)		8.2 (33)	6.8 (28)	
Yes	52.9 (62)	63.4 (77)	**0.209**	91.8 (366)	93.2 (391)	
**Problematic cannabis use**						
No	89.6 (105)	92.2 (112)		83.4 (333)	86.8 (364)	
Yes	10.4 (12)	7.8 (10)	0.084	16.6 (66)	13.2 (55)	
**Polysubstance use^a^**						
No	85.1 (100)	89.7 (110)		78.4 (313)	82.2 (344)	
Yes	14.9 (17)	10.3 (12)	0.127	21.6 (86)	17.8 (75)	
**Any substance use problem^b^**						
No	38.1 (44)	30.5 (37)		0.0 (0)	0.0 (0)	
Yes	61.9 (73)	69.5 (85)	0.156	100 (399)	100 (419)	
**Any use of psychotropic medications**						
No	0.0 (0)	0.0 (0)		83.0 (331)	81.3 (341)	
Yes	100 (117)	100 (122)		17.0 (68)	18.7 (78)	0.045

ESTxENDS: Efficacy, Safety, and Toxicology of ENDS as an aid for smoking cessation. SMD: standardized mean difference; not reported when the variable was not accounted for in the inverse probability of treatment weights. Absolute values are reported.

a Use of ≥2 illicit substances.

b Any of: at-risk alcohol use, problematic cannabis use, or polysubstance use.

Proportions/n and means/standard deviations are calculated with inverse probability of treatment weighting to account for unbalance between treatment groups. Unbalanced characteristics are highlighted in bold.

**Table 2:** Weighted descriptive statistics of medications for the two subsamples of the randomized controlled trial ESTxENDS, Switzerland, 2019–2021

*Medications*	*Subsample with psychiatric problems* *(N=239)*			*Subsample with substance use problems* *(N=818)*		
	*Intervention* *% (n)*	*Control* *% (n)*	*SMD*	*Intervention* *% (n)*	*Control* *% (n)*	*SMD*
**Total,** n	117	122		399	419	
**Antidepressants** (ATC code NO6A)						
No	43.1 (51)	35.7 (44)		89.3 (356)	87.8 (368)	
Yes	56.9 (66)	64.3 (78)		10.7 (43)	12.2 (51)	0.047
**Antipsychotics** (ATC code NO5A)						
No	83.5 (98)	84.5 (103)		96.4 (385)	96.3 (403)	
Yes	16.5 (19)	15.5 (19)		3.6 (14)	3.7 (16)	0.008
**Anxiolytics: benzodiazepine derivates** (ATC code NO5BA)						
No	83.4 (98)	86.2 (98)		95.1 (380)	96.5 (404)	
Yes	16.6 (19)	13.8 (24)		4.9 (19)	3.5 (15)	0.074
**Hypnotics/sedatives** (ATC code NO5C)						
No	84.2 (99)	83.2 (101)		96.9 (387)	96.5 (404)	
Yes	15.8 (18)	16.8 (21)		3.1 (12)	3.5 (15)	0.022
**Hypnotics/sedatives: benzodiazepine derivates** (ATC code NO5CD)						
**No**	**99.1 (116)**	**100 (122)**		**99.4 (397)**	100 (419)	
Yes	0.9 (1)	0.0 (0)		0.6 (2)	0.0 (0)	0.113

ESTxENDS: Efficacy, Safety, and Toxicology of ENDS as an aid for smoking cessation. ATC code: Anatomic Therapeutic Chemical classification. SMD: standardized mean difference; not reported when the variable was not accounted for in the inverse probability of treatment weights. Absolute values are reported. Proportions/n are calculated with inverse probability of treatment weighting to account for unbalance between treatment groups.

**Figure 1:**
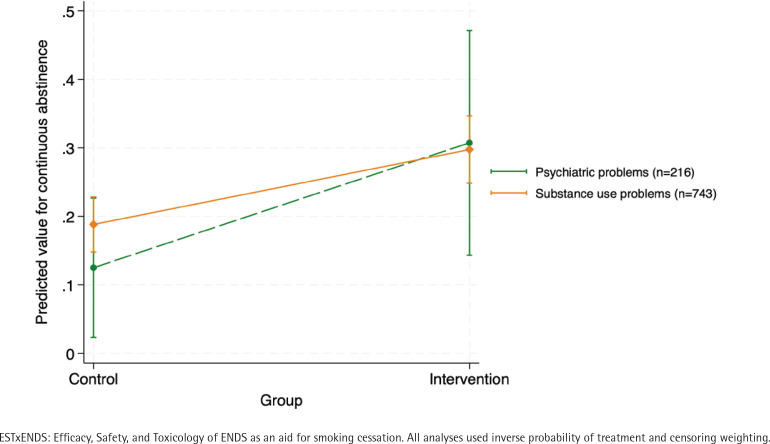
Predictive margins for the interaction effect between group of the randomized controlled trial ESTxENDS and subsample for the primary outcome, Switzerland, 2019-2021

At 6 months, as the primary outcome, we collected data for 216 participants with psychiatric problems (retention rate was 90.4%; n=107 in the intervention group; n=109 in the control group) and 743 participants with substance use problems (retention rate was 90.8%; n=369 in the intervention group; n=374 in the control group). Supplementary file Table S1 provides weighted descriptive statistics for the two subsamples with primary outcome data at 6 months, using IPTW and IPCW. We report the main results in Table 3 and Figure 1.

**Table 3:** Weighted comparisons between groups of the randomized controlled trial ESTxENDS for primary and secondary outcomes, Switzerland, 2019–2021

*Outcomes*	*Total* *n*	*Subsample with psychiatric problems*			
		*Intervention* *% (n)*	*Control* *% (n)*	*RR (95% CI)*	*ARR (95% CI)*
**Primary outcome**					
Continuous abstinence with biochemical validation	215	30.8 (31)	14.8 (14)	2.28 (1.20–4.33)	2.62 (1.40–4.90)
**Secondary outcomes**					
Continuous abstinence without biochemical validation	215	41.3 (44)	15.8 (17)	2.61 (1.50–4.53)	2.95 (1.72–5.07)
Abstinence within previous 7 days with biochemical validation	201	43.1 (44)	15.2 (15)	2.84 (1.58–5.10)	3.05 (1.72–5.42)
Abstinence within previous 7 days without biochemical validation	201	58.8 (59)	20.9 (21)	2.76 (1.75–4.37)	2.96 (1.92–4.55)
		***Subsample with substance use problems***			
**Primary outcome**					
Continuous abstinence with biochemical validation	742	29.0 (107)	18.9 (71)	1.53 (1.17–2.01)	1.57 (1.20–2.04)
**Secondary outcomes**					
Continuous abstinence without biochemical validation	742	37.9 (139)	27.0 (101)	1.41 (1.13–1.75)	1.42 (1.15–1.74)
Abstinence within previous 7 days with biochemical validation	707	42.2 (151)	25.9 (91)	1.63 (1.31–2.03)	1.68 (1.35–2.09)
Abstinence within previous 7 days without biochemical validation	707	58.4 (208)	38.5 (135)	1.52 (1.29–1.78)	1.53 (1.31–1.79)

ESTxENDS: Efficacy, Safety, and Toxicology of ENDS as an aid for smoking cessation. All analyses (crude and adjusted) used inverse probability of treatment and censoring weighting. ARR: adjusted relative risk; calculated adjusting for study site, age, gender, marital status, education level, work status, age first used cigarettes, cigarettes/day, history of quit attempts, and Fagerström score. In addition, adjusted for at-risk alcohol use, problematic cannabis use, and polysubstance use in the subsample with psychiatric problems; and use of psychotropic medications in the subsample with substance use problems.

In the subsample of participants with psychiatric problems, 30.8% in the intervention group had biochemically validated continuous abstinence (primary outcome) from smoking at 6 months in the intervention group versus 14.8% in the control group (weighted adjusted relative risk ARR=2.62; 95% CI: 1.40–4.90). Self-reported continuous abstinence without validation (secondary outcome) from smoking at 6 months was 41.3% in the intervention group and 15.8% in the control group (weighted ARR=2.95; 95% CI: 1.72–4.55). Self-reported 7-day abstinence with validation (secondary outcome) was 43.1% in the intervention group and 15.2% in the control group (weighted ARR=3.05; 95% CI: 1.72–4.42). Self-reported 7-day abstinence without validation (secondary outcome) was 58.8% in the intervention group and 20.9% in the control group (weighted ARR=2.96; 95% CI: 1.92–4.55).

In the subsample of participants with substance use problems, 29.0% had biochemically validated continuous abstinence from smoking at 6 months in the intervention group versus 18.9% in the control group (weighted ARR=1.57; 95% CI: 1.20–2.04). Self-reported continuous abstinence from smoking, without validation at 6 months, was 37.9% in the intervention group and 27.0% in the control group (weighted ARR=1.42; 95% CI: 1.15–1.74). Self-reported 7-day abstinence with validation was 42.2% in the intervention group and 25.9% in the control group (weighted ARR=1.68; 95% CI: 1.35–2.09). Self-reported 7-day abstinence without validation was 58.4% in the intervention group and 38.5% in the control group (weighted ARR=1.53; 95% CI: 1.31–1.79).

In the subsample with psychiatric problems, we found no interaction effect between the treatment group and sex (weighted ARR for the interaction term=0.65; 95% CI: 0.18–2.34). In the subsample with substance use problems, there was a significant interaction effect between the treatment group and sex (weighted ARR for the interaction term=1.81; 95% CI: 1.04–3.16), indicating that the intervention was more effective in women than in men. The result is shown in Supplementary file Figure S1.

We conducted sensitivity analyses that defined participants with missing smoking status at 6 months as smokers (Supplementary file Table S2) and subgroups with different types of psychotropic medication (Supplementary file Table S3) that returned similar results. Supplementary file Table S4 reports exposure to nicotine at 6 months.

## DISCUSSION

The results of adding e-cigarettes to smoking cessation counseling for participants with psychiatric and substance use problems were promising. Participants with psychiatric problems (defined by the use of psychotropic medications) had a significantly increased likelihood of quitting smoking (ARR=2.62; 95% CI: 1.40–4.90). Participants with substance use problems characterized by problematic use of alcohol, cannabis, or polysubstance use also had an increased likelihood of quitting smoking (ARR=1.57; 95% CI: 1.20–2.04). These findings mirror those observed in the primary study, where the ARR for the primary outcome was 1.71 (95% CI: 1.39–2.12) in the entire sample^10^. Our study findings showed even greater benefits of e-cigarettes for people with psychiatric problems using the secondary outcome of self-reported 7-day abstinence (with and without validation). The relative risk (RR) for self-reported 7-day abstinence without validation was 2.96 (95% CI: 1.92–4.55), significantly higher than the corresponding relative risk in the primary study (RR=1.56; 95% CI: 1.37–1.77).

These data confirm the efficacy of e-cigarettes as a valuable cessation tool, as was confirmed in the general population^10,11^ and they suggest the potential benefits of e-cigarettes as cessation tools in vulnerable populations for which we lack comprehensive data^4,12^. E-cigarettes show promise as a tool for addressing the smoking cessation needs of diverse subgroups, including those with psychiatric and substance use problems. We note that although participants in the intervention group were more likely to stop smoking, they did not necessarily quit consuming nicotine.

### Limitations

This study had several limitations. First, the ESTxENDS trial was not powered to analyze subsamples of participants, and the analyses we conducted for this sub-study were not pre-specified. Although our results were statistically significant, confidence intervals were wide, especially for the subsample of participants with psychiatric problems. We suggest future researchers use larger sample sizes to determine e-cigarettes’ effectiveness in different populations. The need for further investigation is highlighted by the substantial effect that the point estimate for participants with psychiatric problems suggests (RR=2.49).

Second, we based our assessment of psychiatric problems on the use of psychotropic medication. Still, we did not know the indication for or duration of medication use (e.g. chronic vs short-term). Although this proxy was used successfully in previous studies^16^, we suggest future researchers undertake a more accurate assessment of psychiatric disorders to validate our study findings. Researchers should also study the effects of the intervention on different types of psychiatric disorders to uncover potential barriers and disparities. However, our subgroup analysis of participants on different types of psychotropic medication showed similar results, although underpowered, suggesting that the heterogeneity of this subsample did not compromise the validity of our conclusions.

Third, randomization did not account for membership in a vulnerable population we analyzed in this study. Though we used a sophisticated strategy (IPTW) to balance the groups better, some variables remained unbalanced (marital status and risky alcohol use in the subsample of participants with psychiatric problems), and there may have been unknown confounders.

Fourth, even though ESTxENDS inclusion criteria were broad, and the trial did not exclude participants with psychiatric disorders, we expect people with severe psychiatric illnesses to volunteer at a lower rate than those in the general population of smokers who want to quit. Future studies should test the feasibility of using e-cigarettes for smoking cessation in people with severe psychiatric illness. Longitudinal studies that track both smoking and e-cigarette use patterns over time and include measures of psychiatric symptomatology and treatment adherence will provide valuable insight into the long-term effectiveness and safety of e-cigarettes as a smoking cessation aid for people with severe psychiatric illness, identify barriers, and capture their unique experiences.

Fifth, our findings may have limited generalizability to non-European populations, as our sample was drawn exclusively from a Swiss setting with specific healthcare systems and smoking cessation support services. Also, our results may not be generalizable to smokers treated in different healthcare settings or who use different smoking cessation methods.

Sixth, simple imputation, which was preferred because of the problems of covariate imbalance, may not fully capture the uncertainty associated with missing values.

Finally, this study lacked long-term follow-up data to assess the sustained effectiveness of the e-cigarette intervention beyond the initial treatment period. Further research is needed to determine whether the observed smoking cessation benefits persist after free product provision ceases.

### Implications

Since smoking exacerbates health disparities in vulnerable populations, smoking cessation efforts should be tailored to meet their needs. Unless we recognize and address the unique barriers faced by people with psychiatric and substance use problems, we cannot make the tobacco control landscape more equitable or expect to improve public health outcomes for all. Given this need, our findings have two key implications for public health and clinical practice. First, they suggest that the disproportionately high smoking rates among individuals with psychiatric and substance use problems could be lowered if e-cigarettes were added to smoking cessation interventions. Second, healthcare professionals should consider recommending e-cigarettes as a potential smoking cessation tool for this population while providing appropriate guidance and support.

## CONCLUSIONS

Our analysis, of a subset of participants in a large smoking cessation trial, suggests that adding e-cigarettes to standard-of-care counseling can increase the likelihood that those with psychiatric and substance use problems would abstain from smoking. We suggest future researchers test our finding in larger studies designed specifically to test the efficacy and safety of smoking cessation interventions in this often-marginalized population.

## Supplementary Material



## Data Availability

Data sharing will start from 14 February 2025, a year after the publication of the main article (doi: 10.1056/NEJMoa2308815). Data can be requested via reto.auer@unibe.ch and will be accessible after approval by the steering committee.
