# Supplementary material for: Efficacy of e-cigarettes for smoking cessation in populations with psychiatric and/or substance use problems: A secondary analysis of a randomized controlled trial
Source: Tob Prev Cessat. 2025 Feb 3;11:10.18332/tpc/199473. doi: 10.18332/tpc/199473 (PMC11788852; doi:10.18332/tpc/199473)
Supplement: Supplementary file 1 [file TPC-11-11-s1.pdf]

Supplementary material

Table S1. Weighted descriptive statistics for the two subsamples of the randomized controlled trial ESTxENDS with data on the primary outcome at 6 months, Switzerland, 2019-2021

		Subsample with psychiatric problems (n=216)			Subsample with substance use problems (n=743)		
		Intervention n=107	Control n=109	SMD	Intervention n=369	Control n=374	SMD
Site[1]							
	Bern	31.1 (33)	37.6 (38)	-	36.3 (134)	35.9 (134)	-
	Geneva	19.5 (21)	24.6 (25)	-	24.6 (91)	24.5 (91)	-
	Lausanne	10.8 (12)	21.3 (21)	-	12.9 (48)	12.6 (47)	-
	St Gallen	16.2 (17)	9.4 (9)	-	13.9 (51)	13.5 (51)	-
	Zurich	22.4 (24)	16.1 (16)	-	12.3 (45)	13.5 (51)	-
Gender[1]							
	Women	49.1 (52)	47.9 (52)	-	48.3 (178)	46.1 (173)	-
	Men	50.9 (55)	52.1 (57)	.023	51.7 (191)	53.9 (201)	.043
Age[2]		40.5 (13.5)	42.5 (12.5)	.160	39.6 (13.5)	41.6 (13.9)	.152
Marital status[1]							
	Single/divorced	66.1 (71)	78.7 (86)	-	73.4 (271)	72.3 (270)	-
	Married	33.9 (36)	21.3 (23)	**.292**	26.6 (98)	27.7 (104)	.025
Level of education[1]							
	Primary	7.5 (8)	6.9 (7)	-	6.6 (24)	8.8 (33)	-
	Secondary	47.0 (50)	41.2 (45)	.149	48.7 (180)	42.3 (158)	.157
	Tertiary	45.5 (49)	51.9 (57)	**.335**	44.6 (165)	48.9 (183)	.115
Work status[1]							
	Employed/training	74.6 (80)	75.6 (82)	-	72.2 (266)	72.2 (270)	-
	Unemployed	25.4 (27)	24.4 (27)	.022	27.8 (103)	27.8 (104)	.001
Age at first use of cigarette[2]		17.5 (3.8)	17.4 (4.6)	.027	17.4 (3.9)	17.3 (3.6)	.014
No. of cigarettes per day[2]		16.4 (8.8)	16.0 (7.4)	.043	16.8 (8.0)	16.9 (7.9)	.020
Tried to quit smoking[1]							
	No	12.8 (14)	17.2 (19)	-	13.5 (50)	15.5 (58)	-
	Yes	87.2 (93)	82.8 (90)	.121	86.5 (319)	84.5 (316)	.058
Fagerström score (0-10)[2]		4.4 (2.3)	3.9 (2.6)	**.204**	4.4 (2.3)	4.3 (2.4)	.023
At-risk alcohol use[1]							
	No	45.8 (49)	37.1 (41)	-	7.2 (26)	6.6 (25)	-
	Yes	54.2 (58)	62.9 (68)	.174	92.8 (343)	93.4 (349)	-
Problematic cannabis use[1]							
	No	89.1 (95)	91.9 (100)	-	84.5 (312)	87.3 (327)	-
	Yes	10.9 (12)	8.1 (9)	.093	15.5 (57)	12.7 (47)	-
Polysubstance use (use of ≥ 2 illicit substances)[1]							
	No	85.4 (91)	89.1 (97)	-	81.1 (299)	82.5 (308)	-
	Yes	14.6 (16)	10.9 (12)	.101	18.9 (70)	17.5 (66)	-
Any substance use problem[1,3]							
	No	36.4 (39)	30.6 (33)	-	0.0 (0)	0.0 (0)	-
	Yes	63.6 (68)	69.4 (76)	.121	100 (369)	100 (374)	-
Any use of psychoactive medications[1]							
	No	0.0 (0)	0.0 (0)	-	81.4 (300)	79.5 (297)	-
	Yes	100 (107)	100 (109)	-	18.6 (69)	20.5 (77)	.050

ESTxENDS: Efficacy, Safety, and Toxicology of END as an aid for smoking cessation; SMD: standardized mean differences; not reported when the variable was not accounted for in the inverse probability of treatment and censoring weights. Absolute values are reported.

[1] Percentages (n) are reported.

[2] Means (standard deviations) are reported.

[3] Any of: at-risk alcohol use, problematic cannabis use, or polysubstance use.

Proportions/n and means/standard deviations are calculated with inverse probability of treatment and censoring weighting to account for unbalance between treatment groups and dropouts. Unbalanced covariates are highlighted in bold.

Table S2. Weighted comparisons between groups of the randomized controlled trial ESTxENDS for primary and secondary outcomes with missing considered as smokers, Switzerland, 2019-2021

Outcome	Intervention group	Control group	Crude relative risk (95% CI)	Adjusted relative risk (95% CI)
	**Subsample with psychiatric problems** (n=239)			
Primary outcome: continuous abstinence with biochemical validation	28.6 (34)	12.5 (15)	2.30 (1.19; 4.41)	2.71 (1.43; 5.14)
Secondary outcome: continuous abstinence without biochemical validation	37.9 (44)	14.6 (18)	2.59 (1.48; 4.55)	2.97 (1.73; 5.10)
Secondary outcome: abstinence within previous 7 days with biochemical validation	38.0 (45)	13.0 (16)	2.93 (1.61; 5.35)	3.21 (1.79; 5.64)
Secondary outcome: abstinence within previous 7 days without biochemical validation	51.2 (60)	18.0 (22)	2.85 (1.78; 4.56)	3.07 (1.98; 4.77)
	**Subsample with substance use problems** (n=818)			
Primary outcome: continuous abstinence with biochemical validation	27.1 (108)	16.8 (70)	1.61 (1.23; 2.13)	1.67 (1.27; 2.18)
Secondary outcome: continuous abstinence without biochemical validation	35.4 (141)	24.4 (102)	1.45 (1.17; 1.81)	1.49 (1.20; 1.83)
Secondary outcome: abstinence within previous 7 days with biochemical validation	38.1 (152)	21.4 (90)	1.78 (1.41; 2.23)	1.82 (1.45; 2.28)
Secondary outcome: abstinence within previous 7 days without biochemical validation	52.8 (211)	32.5 (136)	1.63 (1.37; 1.93)	1.65 (1.40; 1.94)

ESTxENDS: Efficacy, Safety, and Toxicology of END as an aid for smoking cessation; CI: confidence interval.

All analyses (crude and adjusted) used inverse probability of treatment weighting. The adjusted relative risks were calculated adjusting for study site, age, gender, marital status, level of education, work status, age at first cigarette use, no. of cigarettes per day, quit attempts, and Fagerström score. In addition, we adjusted for at-risk alcohol use, problematic cannabis use, and polysubstance use in the subsample with psychiatric problems; and use of psychotropic medications in the subsample with substance use problems.

Table S3. Weighted comparisons for subsamples with different types of psychotropic medications between groups of the randomized controlled trial ESTxENDS for primary outcome, Switzerland, 2019-2021

Outcome	n	Intervention group	Control group	Crude relative risk (95% CI)
		**Subsample with antidepressants**		
Primary outcome: continuous abstinence with biochemical validation	136	30.1 (20)	11.0 (8)	2.73 (1.18; 6.31)
		**Subsample with antipsychotics**		
Primary outcome: continuous abstinence with biochemical validation	46	30.2 (8)	11.1 (2)	2.72 (0.52; 14.33)
		**Subsample with anxiolytics benzodiazepine derivates**		
Primary outcome: continuous abstinence with biochemical validation	37	43.8 (9)	21.9 (4)	2.00 (0.44; 9.05)
		**Subsample with hypnotics/sedatives including benzodiazepine derivates**		
Primary outcome: continuous abstinence with biochemical validation	34	14.7 (3)	10.8 (2)	1.36 (0.19; 10.06)

ESTxENDS: Efficacy, Safety, and Toxicology of END as an aid for smoking cessation; CI: confidence interval.

Analyses used inverse probability of treatment and censoring weighting.

Table S4. Previous seven-day exposure to nicotine and tobacco products at 6 months in participants of the randomized controlled trial ESTxENDS, Switzerland, 2019-2021

	**Subsample psychiatric problems** (n=190)		**Substance substance use problems** (n=688)	
	Control group (n=91)	Intervention group (n=99)	Control group (n=338)	Intervention group (n=350)
**No tobacco cigarettes users (tobacco abstainers)**	23%	54%	40%	58%
**No e-cigarette and no tobacco cigarettes (tobacco and nicotine abstainers)**	22% (20)	9% (9)	37% (125)	11% (38)
with NRT	3% (3)	0% (0)	2% (8)	0% (0)
with smoking cessation medication	1% (1)	0% (0)	0% (0)	0% (0)
**e-cigarette users and no tobacco cigarettes (exclusive e-cigarette users)**	1% (1)	44% (44)	3% (10)	47% (165)
without nicotine in ENDS	0% (0)	6% (6)[1]	1% (3)[2]	8% (29)[3]
with nicotine in ENDS	1% (1)	38% (38)	2% (7)	39% (136)
with NRT	0% (0)	0% (0)	0% (0)	0% (0)
**Tobacco cigarettes users**	77% (70)	46% (46)	60% (203)	42% (147)
**e-cigarette and tobacco cigarettes users (dual users)**	5% (5)	17% (17)	2% (8)	18% (63)
without nicotine in ENDS	3% (3)[4]	1% (1)	1% (4)[5]	2% (7)[6]
with nicotine in ENDS	2% (2)	16% (16)	1% (4)	16% (56)
with NRT	0% (0)	1% (1)	0% (0)	1% (2)
**No e-cigarette and tobacco cigarettes users (smokers)**	71% (65)	29% (29)	58% (195)	24% (84)
with NRT	2% (2)	3% (3)	3% (9)	1% (3)
with smoking cessation medication	1% (1)	0% (0)	1% (2)	0% (0)

Percentage (n) are reported.

ESTxENDS: Efficacy, Safety, and Toxicology of END as an aid for smoking cessation; NRT: Nicotine replacement therapy (i.e., nicotine gum, nicotine inhaler, nicotine lozenge, nicotine patch, and nicotine oral spray used in past 24 hours).

[1] 3 missing values imputed as no nicotine use; [2] 1 missing value imputed as no nicotine use; [3] 9 missing values imputed as no nicotine use; [4] 3 missing values imputed as no nicotine use; [5] 3 missing values imputed as no nicotine use; [6] 4 missing values imputed as no nicotine use.

These results suggest that participants in the intervention group stopped smoking cigarettes, but continued to use e-cigarettes, likely with nicotine, but these descriptive findings should not be overinterpreted.

Figure S1. Predictive margins for the interaction effect between groups of the randomized controlled trial ESTxENDS and sex for the primary outcome in the subsample with substance use problems, Switzerland, 2019-2021 (n=743)

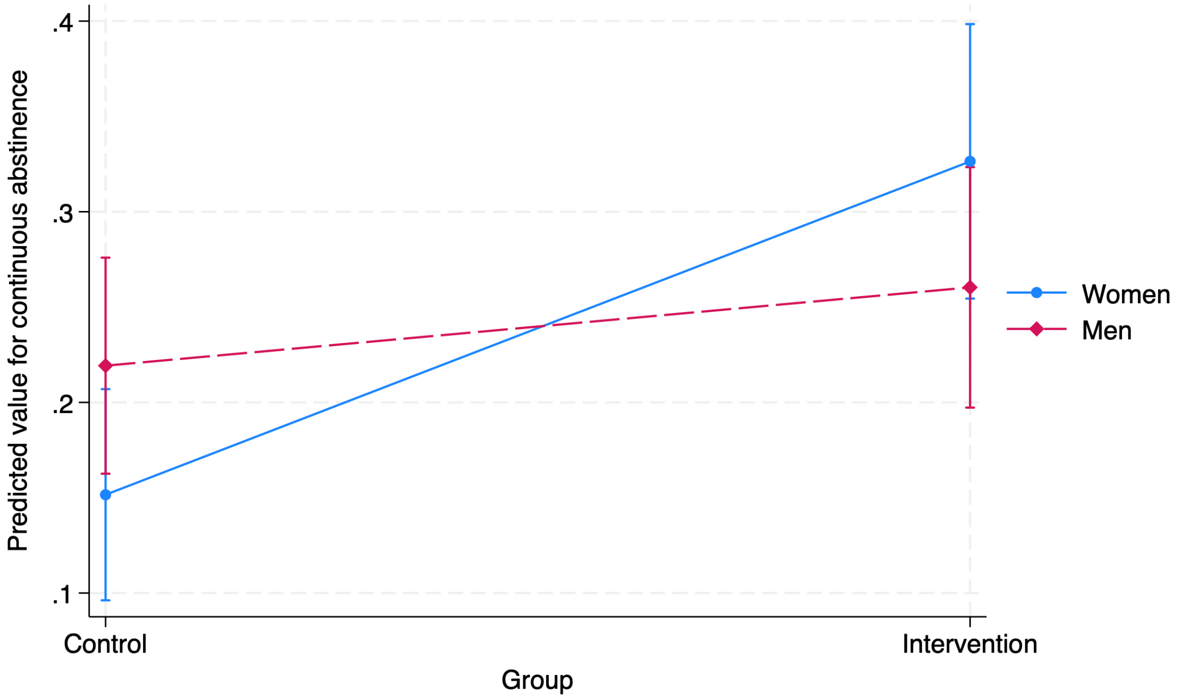

ESTxENDS: Efficacy, Safety, and Toxicology of END as an aid for smoking cessation.

The analysis used inverse probability of treatment weighting and the adjusted relative risks were calculated adjusting for study site, age, gender, marital status, level of education, work status, age at first cigarette use, no. of cigarettes per day, quit attempts, Fagerström score, and use of psychotropic medications.

© 2025 Baggio S. et al.
